# Assessing the genetic diversity of rice originating from Bangladesh, Assam and West Bengal

**DOI:** 10.1186/s12284-015-0068-z

**Published:** 2015-12-01

**Authors:** Anthony J. Travis, Gareth J. Norton, Sutapa Datta, Ramendra Sarma, Tapash Dasgupta, Filipe L. Savio, Malcolm Macaulay, Peter E. Hedley, Kenneth L. McNally, Mahmud H. Sumon, M. Rafiqul Islam, Adam H. Price

**Affiliations:** Institute of Biological and Environmental Sciences, University of Aberdeen, Aberdeen, AB24 3UU UK; Department of Genetics, ICAR-Indian Agricultural Research Institute, New Delhi, India; Department of Plant Breeding and Genetics, Assam Agricultural University, Jorhat, 785013 Assam India; Department of Genetics and Plant Breeding, Calcutta University, 35 B.C. Road, Kolkata, 700 019 West Bengal India; Luiz de Queiroz College of Agriculture, University of São Paulo, Avenida Pádua Dias, 11, Bairro Agronomia, Piracicaba, São Paulo Brazil; Cell & Molecular Sciences, The James Hutton Institute, Invergowrie, Dundee, DD2 5DA UK; International Rice Research Institute (IRRI), DAPO 7777, Metro Manila, 1031 The Philippines; Department of Soil Science, Bangladesh Agricultural University, Mymensingh, Bangladesh

**Keywords:** Rice, SNP, Aus, Boro, Genotype, Ecotype

## Abstract

**Background:**

Genetic diversity among rice cultivars from Bangladesh and North East India was assessed using a custom 384-SNP microarray assay. A total of 511 cultivars were obtained from several sources, choosing landraces likely to be from the *aus* subpopulation and modern *improved* cultivars from Bangladesh. Cultivars from the OryzaSNP set and Rice Diversity Panel 1 (RDP1) were also included for reference.

**Results:**

The population analysis program STRUCTURE was used to infer putative population groups in the panel, revealing four groups: *indica* (76 cultivars)*, japonica* (55) and two distinct groups within the *aus* subpopulation (*aus-1* = 99, *aus-2 =* 151). Principal Component Analysis was used to confirm the four population groups identified by STRUCTURE. The analysis revealed cultivars that belonged to neither *aus-1* nor *aus-2* but which are clearly *aus* based on the combined probabilities of their membership of the two *aus* groups which have been termed *aus-admix* (96). Information obtained from the panel of 511 cultivars was used to assign rice groups to 74 additional landraces obtained from Assam and West Bengal. While both the *aus-1* and *aus-2* groups were represented approximately equally in India, *aus-2* (which includes cultivar N 22) was more common in Bangladesh, but was not found at all in West Bengal.

**Conclusions:**

Examining the distribution of landrace names within the*aus-1* and *aus-2* groups suggests that *aus-1* is associated with the term “boro”, a word used to describe a winter growing season in Bangladesh and Assam. The information described here has been used to select a population of 300 cultivars for Genome Wide Association studies of the *aus* rice subpopulation.

**Electronic supplementary material:**

The online version of this article (doi:10.1186/s12284-015-0068-z) contains supplementary material, which is available to authorized users.

## Background

Rice (*Oryza sativa* L.) has been cultivated as a crop for at least 8000 years (Maclean *et al.,*^2002^) and currently 50 % of the world’s population is dependent on rice as their staple diet. Within rice germplasm there is considerable genetic diversity that reflects its domestication and long breeding history and, most notably, two subspecies of rice are recognised: *indica* and *japonica*. There are a number of different theories about the domestication of rice. Molina *et al.* (2011) present evidence that rice was domesticated from *Oryza rufipogon* at a single point in time while in the review by Khush (1997) independent origins of domestications for *japonica* (in China) and *indica* (in North East India) are suggested. Recently, Huang *et al.* (2012) argue that *japonica* rice was domesticated from a population of *O. rufipogon* in southern China, but that the *indica* sub-species originated from a crossing of *japonica* and wild rice as rice cultivation spread into South and South East Asia.

The first widely adopted molecular classification of rice groups was based on isozyme analysis (Glaszmann, 1987), in which six varietal groups were identified. These are usually referred to as: I = *indica*, II = *aus*, III = *ashina*, IV = *rayada*, V = *aromatic*, VI = *japonica* (from Glaszmann, 1987 as modified by, for example, Wang *et al.,*^2013^). Subsequent DNA analysis has identified five subpopulation of rice within the two *Oryza* sub-species. The *aus* subpopulation was identified within *indica*, and the *temperate*, *tropical* and *aromatic* subpopulations were identified within *japonica* (Garris *et al.,*^2005^). These five subpopulations are now commonly used to describe rice cultivars.

Glaszmann’s group II (*aus*), which was originally considered to be exclusive to South and West Asia, was described as having a short life cycle and grown under a range of conditions from fully irrigated to upland (Glaszmann, 1987). This work suggested that group II rice encompasses the aus ecotype but also includes some boro ecotype rice. Normally, the term ‘boro’ refers to a growing season in Bangladesh and Assam during December-May (GRiSP, 2013) and also to genetically diverse cultivars grown during this season (Parsons *et al.,*^1999^).”aus/ahu” refers to a growing season in Bangladesh and Assam during April-August (GRiSP, 2013) and also to the rice cultivars grown during this season, which are broadcast sown. These aus/ahu rice cultivars are insensitive to photoperiod and are drought tolerant (Khush, 1997). The geographical term aus and the genetic term *aus* can cause confusion because there is considerable overlap between *boro* and *aus* cultivars at the genetic level, which means the geographical distinction between them is blurred. Most importantly, cultivars grown during the boro or aus seasons may not all be genetically ‘*aus’* cultivars. The recently announced 3,000 rice genomes project (Li et al. 2014) refers to a group of *aus/boro* genotypes, which is the genetic group normally referred to as *aus*. A total of 208 accessions out of the 3,000 rice genomes are classified as *aus/boro,* based on 200,000 SNP markers. A subsequent phylogenetic analysis of all 3,000 genome sequences using 376,000 SNP markers also revealed a genetically related group called *aus* (Alexandrov *et al.,*^2015^). In this paper, we refer to genotypes from this group as ‘*aus’* using italics to refer to genetic terms and ‘aus’ in normal font for similar geographic or ecotype terms.

A recent analysis of 409 rice cultivars indicated that lines from the *aus* group originate predominantly from areas in Bangladesh and India (Ali *et al.,*^2011^). The genetic diversity of the aus and boro ecotypes is large and includes a number of cultivars known for their adaptation to different environments. Cultivar FR 13A, for example, is the flood tolerant donor of the submergence tolerance gene *Sub1* (Xu *et al.,*^2006^); Kasalath is the efficient phosphorus uptake donor of the phosphorus starvation tolerance gene *Pstol1* (Gamuyao *et al.,*^2012^); Dular is a rice cultivar that has increased drought resistance, associated with greater root length and root density (Henry *et al.,*^2011^); Rayada also has a large root length and high root density (Henry *et al.,*^2011^); Black Gora is a rice cultivar with high seedling vigour (Redoña and Mackill, 1996) and deep roots (Shrestha *et al.,*^2014^; Al-Shugeairy *et al.,*^2014^) and; N 22 is a heat tolerant rice cultivar (Jagadish *et al.,*^2008^).

A number of previous studies have examined the genetic diversity of rice cultivars from Bangladesh (Parsons *et al.,*^1999^; Sajib *et al.,*^2012^; Hassan *et al.,*^2012^). However these studies involved relatively small numbers of genetic markers. Studies of global populations of rice (Ali *et al.,*^2011^) using a larger number of markers to capture global rice genetic diversity are limited by the relatively small number of cultivars obtained from specific geographic regions. The aim of this study is to examine the genetic variation of about 500 landraces from the aus and boro ecotypes of rice that fall within the *aus* genetic group of rice based on 384 SNP markers and also use the information obtained to identify the genetic background of field-grown cultivars collected from farmers in West Bengal and Assam in India. The cultivars investigated here will be used to establish a panel of *aus* cultivars for a subsequent genome wide association (GWA) mapping study within a genetically and geographically distinct group of rice genotypes.

## Results

**Fig. 1:**
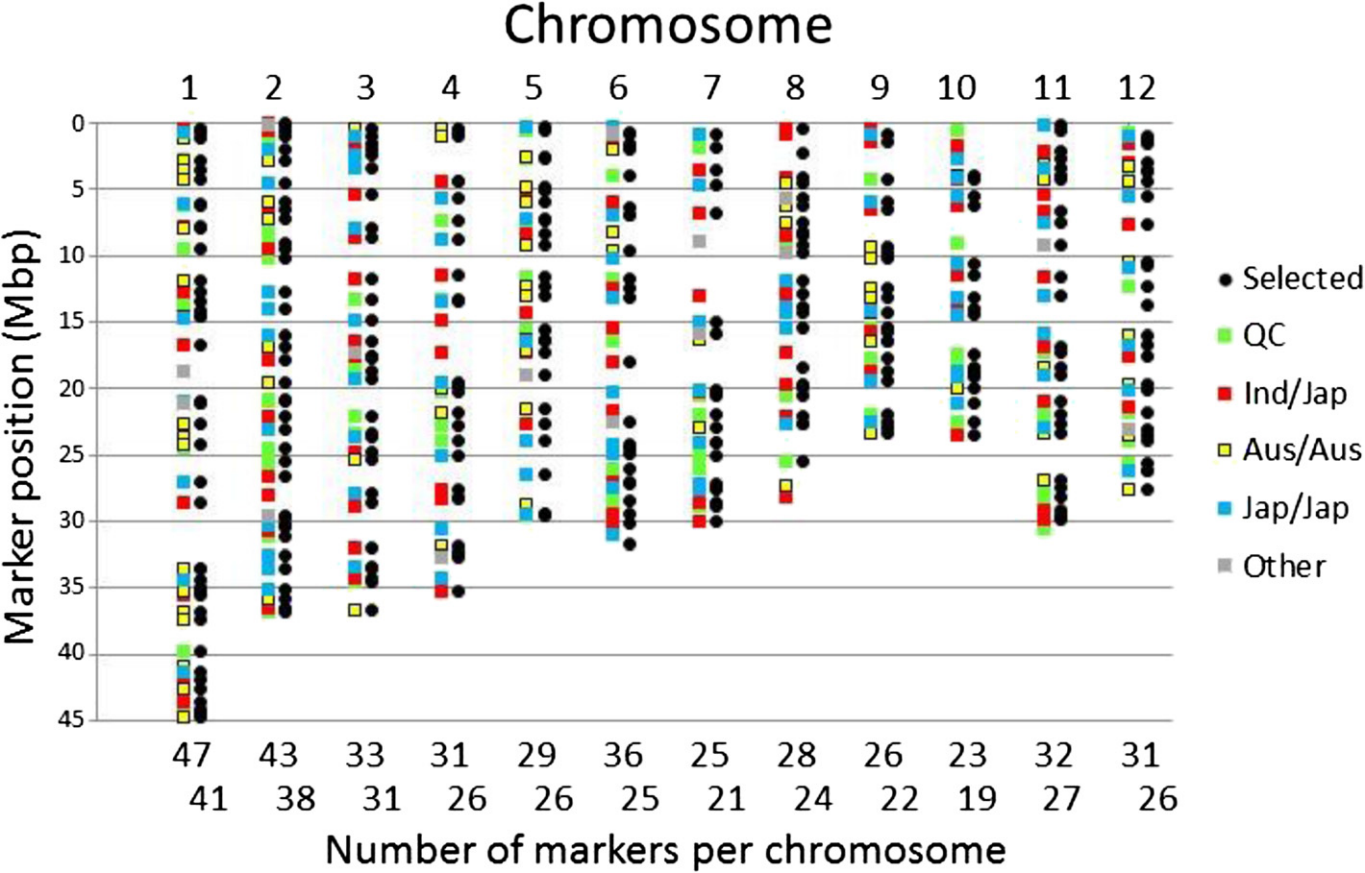
Physical position of the 384 SNPs according to the category of their predicted polymorphic discrimination. Based on data from the Rice Diversity website, where QC is the “Quality Control” set which can “Assign accessions into one of five *O. sativa* subgroups” and at the bottom of each chromosome is the number of markers on that chromosome (Selected SNPs, to the right, are the 326 SNP remaining for genetic analysis after filtering of poor quality SNP-calls on any one plate used in this study)

The design of the SNP chip was intended to achieve an approximately even spread of markers across the entire rice genome. Before the analysis, data for 58 of the 384 SNPs were removed because they displayed a high degree of heterozygosity on some microarray plates but not on others and were therefore considered to be unreliable. This resulted in a total of 326 markers included in the analysis which remain approximately evenly placed across the rice genome although some gaps are apparent, for example at the middle of chromosomes 4 and 7, and also at the top of chromosome 10 (Fig. 1).

**Fig. 2:**
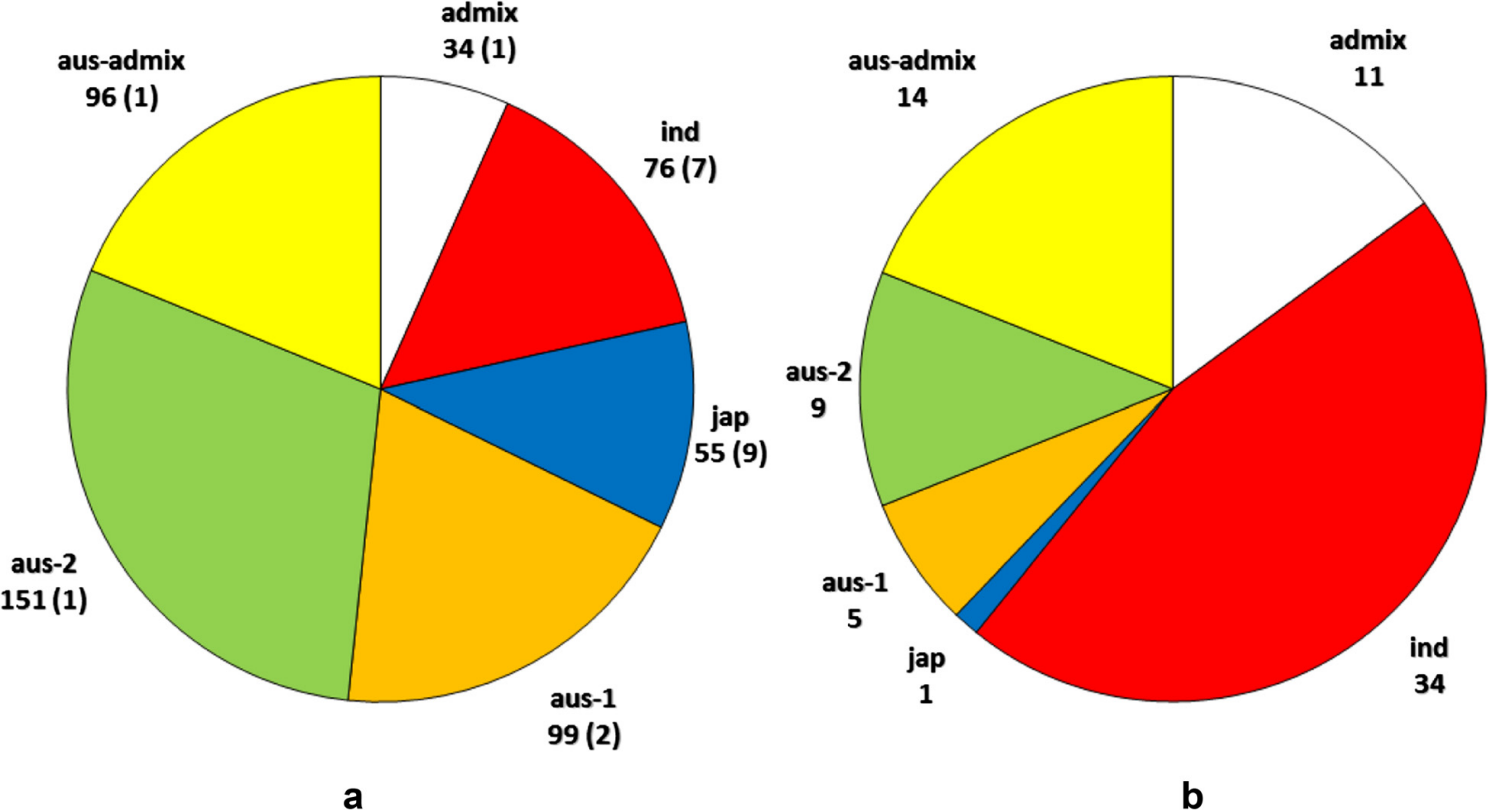
Distribution of cultivars in (**a**) Panel A (511 cultivars) and (**b**) Panel B (74 cultivars). The cultivars in Panel **b** were obtained from Assam Agricultural University and Calcutta University. Cultivars were assigned to rice groups on the basis of exemplars with > = 80 % probability using STRUCTURE (white = *admix*, red = *indica*, blue = *japonica*, orange = *aus-1*, green = *aus*-2, yellow = *aus-admix*; Numbers in brackets = OryzaSNP cultivars)

The STRUCTURE population analysis of Panel A (511 cultivars) indicated that there were 4 distinct population groups present using the Evano Delta-K method as shown in Additional file 1: Figure S1 (Online Resource 4). The group memberships for each cultivar in Panel A are summarised in Additional file 2: Figure S2 (Online Resource 5). Based on *a priori* knowledge of the rice subpopulations that the exemplar cultivars belonged to shown in Additional file 3: Table S1 (Online Resource 1) one of the STRUCTURE groups was determined to be *japonica (jap)* and another to be *indica* (*ind)*. However, the two remaining groups both included cultivars that, in previous studies, have been shown to belong to the *aus* subpopulation and were therefore designated *aus-1* and *aus-2*. Cultivars were allocated to four groups: *ind* (indica), *jap* (japonica), *aus-1* and *aus-2.* Some of the remaining cultivars were clearly from the *aus* subpopulation based on the sum of their probabilities of membership to the two *aus* groups and these have been classified as *aus-admix.* All other cultivars are considered as *admix.* The largest number of cultivars in Panel A (OryzaSNP cultivars excluded) belong to *aus-2* (150 cultivars) with the *japonica* group having only 50 cultivars (Fig. 2a).

**Fig. 3:**
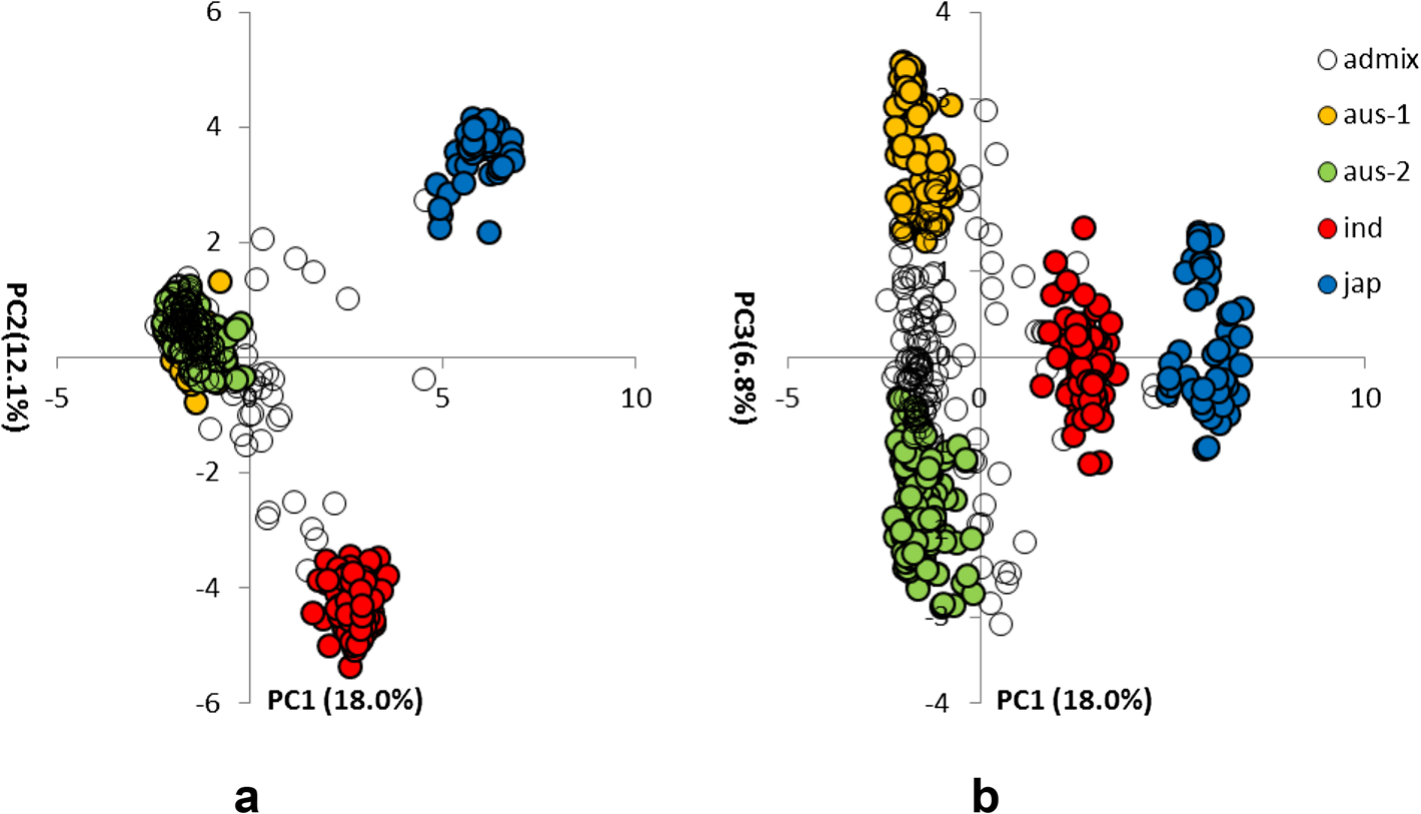
Principal Component Analysis of 511 cultivars in Panel A. (**a **) PC1/PC2 showing three clusters corresponding to the main rice groups *indica, japonica, aus*; (**b**) PC1/PC3 showing sub-groups within the *aus* cluster. Cultivars were assigned to rice groups on the basis of exemplars with > = 80 % probability using STRUCTURE (red = *indica*, blue = *japonica*, orange = *aus-1*, green = *aus*-2, white = *admix* (either *aus-admix* or *admix)*

**Fig. 4:**
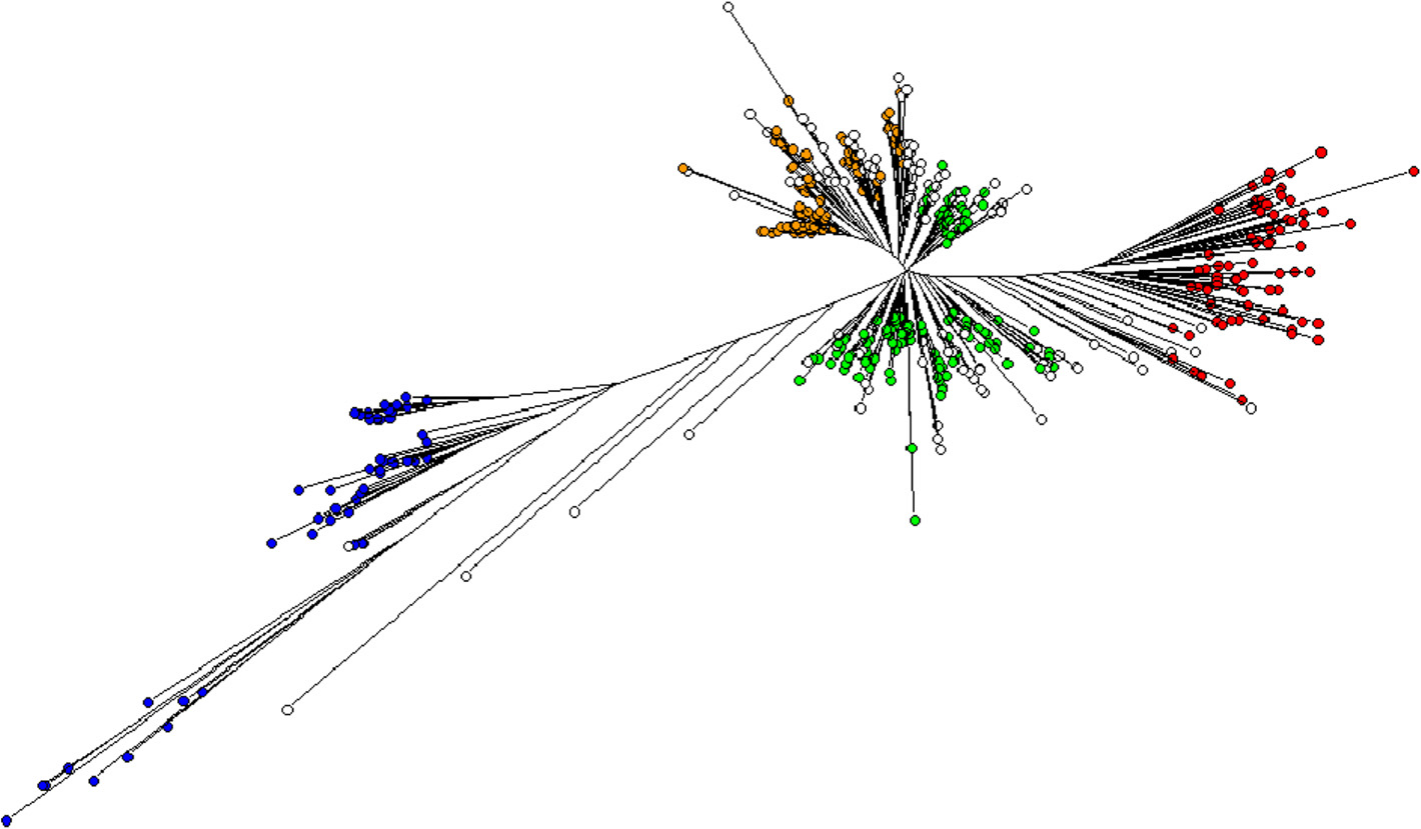
Neighbour-Joining tree (50 % consensus of 100 bootstraps) of 511 cultivars in Panel A. Cultivars were classified into rice groups based on > = 80 % probability of STRUCTURE group membership: *Japonica* (blue), *indica* (red), *aus* group1 (orange), *aus* group2 (green), *aus-admix* and *admix* (white) with < 80 % probability of belonging to a single designated STRUCTURE group. A nexml file of this NJ tree that can be visualised in Dendroscope is provided as Additional file 6

Using PCA it was possible to separate out the 511 cultivars in Panel A into groups that support the STRUCTURE analysis (Fig. 3). Both PCA axis 1 and PCA axis 2 separate the *japonica*, *indica* and *aus* subpopulations (Fig. 3a). It was also possible to separate out the *aus* cultivars into the two groups when PCA 1 was plotted against PCA 3 (Fig. 3b). The upper group in this PCA plot was arbitrarily named “*aus-1*” (orange in Fig. 3) and the lower one “*aus-2*” (green in Fig. 2). There are 96 cultivars that lie directly between the *aus-1* and *aus-2* groups in Fig. 3b which have been classified as *aus-admix.* These do not belong exclusively to either the *aus-1* or *aus-2* group based on a threshold of 80 % probability of their group membership in the STRUCTURE analysis, but the combined probabilities for their *aus-1* and *aus-2* group memberships exceeds the 80 % threshold, indicating that these are *aus* cultivars. The observation that four distinct groups of cultivars were inferred by STRUCTURE is supported by the NJ tree produced from a multi-FASTA alignment of the Panel A SNP data (Fig. 4). The *indica* and *japonica* groups are distinct while the *aus-1* and *aus-2* groups broadly separate from each other. A “nexml” format file of the NJ tree shown in Fig. 4 that can be visualised using Dendroscope is provided as Online Resource 6.

**Fig. 5:**
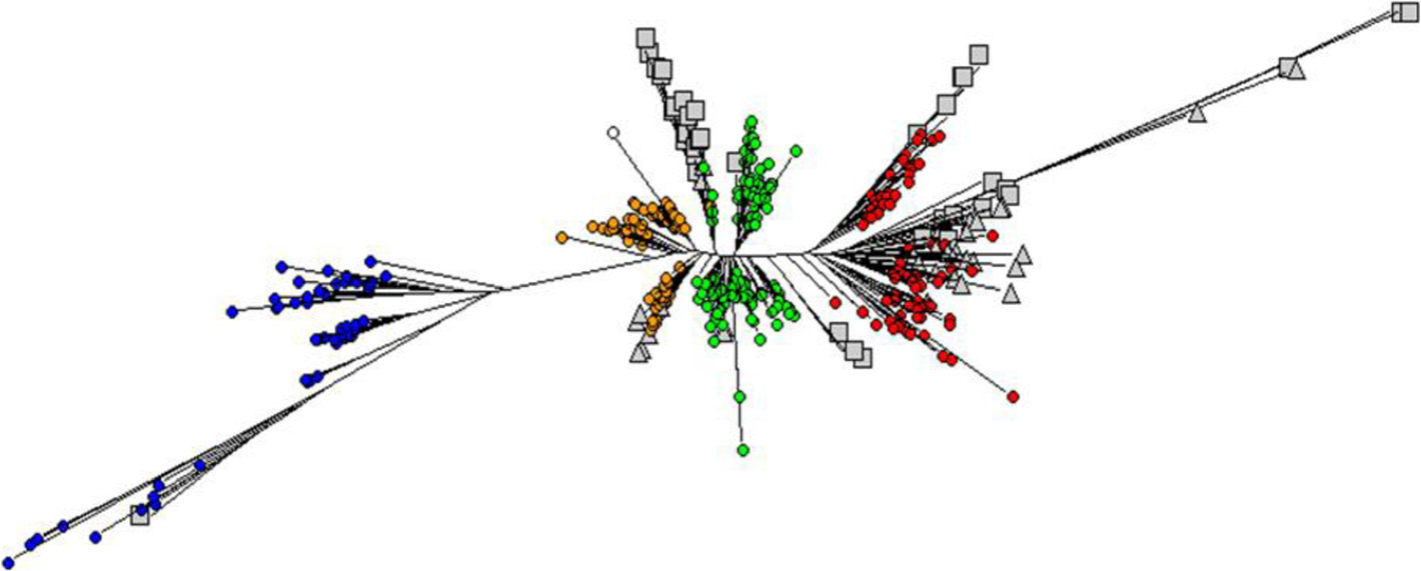
Neighbour-Joining tree (50 % consensus of 100 bootstraps) of 74 cultivars in Panel B + training set. The Assam and West Bengal cultivars in Panel B were classified into rice groups using a training set with ≥ 80 % probability of STRUCTURE group membership selected from all 511 AWD cultivars. Training set: *Japonica* (blue), *indica* (red), *aus* group1 (orange), *aus* group2 (green), *admix* (white), Assam (grey circles), West Bengal (grey squares). A nexml file of this NJ tree that can be visualised in Dendroscope is provided as Additional file 7

Cultivars definitively belonging to one of the four identified rice groups (*indica*, *japonica*, *aus-2* or *aus-1*) at 80 % probability were selected to create a STRUCTURE ‘training’ set of cultivars with pre-defined populations that allowed an assessment to be made about the genetic composition of landraces and improved cultivars obtained from Assam and West Bengal (Panel B). The results are illustrated by the NJ tree shown in Fig. 5. The majority of the West Bengal landraces were identified as *indica* (23 out of 35) as are the two landraces from both Orissa and Bihar, while four are *aus-2*, six are *aus-admix* and three are *admix* (one a *japonica*-like and two *indica*-like admix based on where they cluster in the NJ tree). No West Bengal landraces were identified as either *japonica* or *aus-1*. For the Assam landraces: 12 out of 39 were *indica*, one was *japonica*, nine were *aus-1*, one was *aus-2*, eight were *aus-admix* and eight were *admix* (three *japonica*-like, three *aus*-like and two *indica*-like based on their position in the NJ tree).

## Discussion

Many molecular methods have been developed to assess genetic diversity and the technology improves at a rapid rate. In the study of rice diversity, the first systematic molecular approach was that of Glaszmann (1987). During the 1990s RFLP (e.g. Wang et al. 1995) and PCR-based methods such as RAPD (e.g. Virk *et al.,*^1996^), AFLPs (Virk *et al.,*^2000^) and microsatellite markers (Panaud *et al.*^1996^) were developed. Since the availability of large amounts of sequencing information, such as that provided by the OryzaSNP project (McNally *et al.,*^2009^), highly-multiplexed SNP assay methods have been developed as techniques based on resequencing are beginning to emerge (Huang et al. 2010, Ebana et al. 2010). This study exploits the work led by the activities of the Rice Diversity Project (www.ricediversity.org; Thomson et al. 2012), to develop a targeted 384-SNP BeadXpress assay which is able to differentiate rice cultivars on a genetic basis into the known rice diversity subpopulations. Here, we carefully selected a combination of known SNP-markers to achieve two objectives: First to obtain good classification of rice cultivars into the recognised rice subpopulation, but also to have high resolution within the *aus* subpopulation of the *indica* subspecies. This was achieved by having evenly distributed SNPs throughout the genome (Fig. 1), especially SNPs that were predicted to differentiate *aus* cultivars as revealed in the 44 k SNP data (Zhao *et al.,*^2011^). While several of the SNPs could not be used because they were non-polymorphic or provided low quality data, 326 SNPs were available for diversity analysis. The number of SNPs which were polymorphic within *indica*, *japonica* and *aus* accessions (as classified here) was 191, 229 and 142 respectively.

The results clearly indicate that our custom designed SNP assay achieved its primary objective of being able to correctly allocate known rice cultivars to the *indica*, *aus*, and *japonica* subpopulation (note that too few *temperate japonicas* were included in Panel A to allow subdivision of the *japonica* subspecies into two groups and, likewise, too few aromatic cultivars were included to allow that group to be recognised). Of all the exemplar cultivars used here with rice subpopulation known *a-priori* only one was not correctly allocated to its recognised rice group: the OryzaSNP cultivar Minghui 63, which *was* recognised as an *indica*, but at less than the 80 % threshold we used and was therefore classified as an *admix* including 20 % *aus-2* group membership. Regarding the secondary objective of the SNP assay design, it is also clear that high resolution has been achieved within the *aus* subpopulation of Panel A because two distinct groups of *aus* cultivars were observed. This is the first time, to our knowledge, that a genetic study of rice has revealed such population structure within the *aus* subpopulation of rice. We speculate that this is due to a good choice of SNP-markers in our array design and because of the large number of accessions selected that were revealed to be *aus* (345 of the 511 cultivars used here). It is interesting to note that this subdivision of the *aus* subpopulation was not detected in the 3 K rice genomes project using 20 million SNPs, but only 208 *aus* cultivars (Alexandrov *et al.,*^2015^).

Examining the classification of cultivars into the rice groups presented in Additional file 3: Table S1 (Online Resource 1) indicates that some cultivars that are named “aus” are not genetically *aus*. Thus 10 cultivars with the term “aus” either at the start or end of their name are *indica* and two are *japonica*. This presumably reflects the distinction between the genetic use of the term and the geographic use discussed earlier. Of the 38 cultivars selected from the Assam Rice Collection (these are 6,630 accessions collected between 1965 and 1972 and sent to IRRI (Singh and Singh, 2000)), one was a *japonica*, two were *indica*, seven were admix, 14 were *aus-2*, six were *aus-1* while seven were *aus-admix*. Thus the great majority of cultivars we selected from this collection appear to be *aus* in nature.

Examining the allocation of *aus* cultivars into the two *aus* sub-groups reveals that some well-characterised rice cultivars appear in separate *aus* sub-groups: The drought and heat tolerant cultivar N 22 (Lenka *et al.,*^2011^; Jagadish *et al.,*^2010^) appears in *aus-2,* while FR 13 (the donor of the *Sub1* submergence tolerance gene (Xu *et al.,*^2006^)), Rayada (a Bangladesh deep water rice used in Garris *et al.* (2005)) and Kasalath (the donor of *Pstol*, the phosphorus starvation tolerance gene (Gamuyao *et al.,*^2012^)) all appear in *aus-1*. Other well-characterised *aus* cultivars are classified as *aus-admix* in this study, including Black Gora (a deep rooted check cultivar used by Shrestha *et al.* (2014)), Dular (a donor of drought tolerance) and the recently sequenced *aus* reference cultivar DJ 123 (Schatz *et al.,*^2014^).

The analysis of the 511 cultivars in Panel A provided a training set that revealed an insight into the genetic composition of the 74 improved varieties and landraces from Bangladesh. Most noticeable is the fact that the improved variety BR 16 is an *aus-2* cultivar whereas the others (BR3, BR6, BRRI Dhan 28, 29, 45 and 47, BINA Dhan 5, 6, 8 and 50 and Iratom 24) are all *indica* as might be expected.

It is interesting to note that the landraces which can be allocated to specific regions (276 from Bangladesh, 77 from Assam (including 39 from Panel B and 38 from the Assam Rice Collection) and 31 from West Bengal), are not equally allocated into the rice subpopulations of *indica*, *japonica* and *aus*. The *japonicas* appear to be absent in cultivars selected from West Bengal (none) and are rare in cultivars from Assam (one), but *japonicas* are not uncommon in Bangladesh (32/276). Also potentially interesting is the relative rarity of *aus-1* in Bangladesh (61 *aus-1*, 109 *aus-2* and 49 *aus-admix*) and Assam (1 *aus-1*, 9 *aus-2* and 8 *aus-admix*), and the absence of *aus-2* in cultivars from West Bengal (4 *aus-1* and 6 *aus-admix*). Of the 168 landraces from India that are not part of the Assam Rice Collection, *aus-1* and *aus-2* are approximately equally represented (32 *aus-1*, 28 *aus-2* and 38 *aus-admix*). This suggests that the *aus-1* and *aus-2* sub-groups are geographically distinct, and might indicate that *aus-1* originates from West Bengal.

A final observation is that the 60 cultivars used here whose name contains the term “boro” (all but 9 from Bangladesh) are predominantly *aus-1*. Of these “boro”-named landraces, 34 are *aus-1*, 13 are *japonica*, six are *indica*, four are admix, two are *aus-admix* but only one is *aus-2*. In fact, more than half of the *aus-1* cultivars from Bangladesh have the term “boro” in their names, but they account for less than 16 % of all the *aus* cultivars from Bangladesh studied here. This strongly suggests that selection of cultivars suitable for the boro growing season has caused a differentiation between the *aus-1* and *aus-2* sub-groups identified in this study and that *aus-1* is the genetic type of aus suited to production during the boro season.

## Conclusions

Rice cultivars named by ecotype or growing season are not always of similar genotype and at least two geographically distinct groups of the *aus* genotype exist within the *aus* subpopulation of rice.

## Methods

### Plant material

Two different panels of rice cultivars were used in this study: Panel A was selected to allow the development of a GWA mapping population focused on *aus* cultivars from Bangladesh and North East India and consisted of 511 cultivars from a number of sources: A total 411 accessions were selected from the IRRI gene-bank. These were selected because they originate (or were collected) from either Bangladesh or India and are considered to be *aus* cultivars. In addition to these cultivars, 31 *aus* accessions from Bangladesh or India were selected from the Rice Diversity Panel 1 (Zhao *et al.,*^2011^). A total of 27 known *aus* accessions originating from Bangladesh or India were also obtained from the USDA core rice collection (Yan *et al.,*^2010^). A further 41 cultivars were collected by Bangladesh Agricultural University from sources in Bangladesh, including both landraces and *improved* cultivars. In addition to these cultivars, all of which originate from the North East of India or Bangladesh, Panel A also included 19 accessions from the OryzaSNP panel (McNally *et al.,*^2009^), which were selected to represent the wider genetic diversity of rice. Panel B was selected to provide information about the genetic diversity of a further 74 landraces from the same region: 35 cultivars were collected by Calcutta University from West Bengal (31), Bihar (2) and Orissa (2) in India; 39 cultivars were collected by Assam Agricultural University from Assam in India. A full list of the rice cultivars selected for Panel A, including the germplasm source and country of origin or collection, is presented in Additional file 3: Table S1 (Online Resource 1); A full list of the cultivars selected for Panel B is presented in Additional file 4: Table S2 (Online Resource 2).

### DNA extraction

The 511 cultivars selected for Panel A were grown from seed under controlled conditions and DNA was then extracted from fresh leaf tissue using the DNeasy Plant kit (Qiagen). DNA from the 74 cultivars from Assam and West Bengal selected for Panel B was extracted from dried leaf tissue using a modification of the method described by Deshmukh *et al.* (2007) to include a genomic DNA precipitation step.

### Genotyping

A 384 SNP Illumina GoldenGate array was designed by combining SNP probes selected from data available at the Rice Diversity website (http://ricediversity.org/data/): 83 probes were selected from RiceOPA1.0 (Quality Control), 100 from RiceOPA2.1 (*indica/indica*), 9 from RiceOPA3.1 (*indica/japonica*), 74 from RiceOPA4.0 (*japonica/japonica*), 9 from RiceOPA5.0 (*indica/O. rufipogon*), 4 from RiceOPA6.0 (*japonica/O. rufipogon*) and 105 from RiceOPA7.0 (*indica/japonica*). The aim of combining these probes was to produce a probe-set with a mixture of abilities to detect polymorphism between widely different accessions such as the *indica* and *japonica* sub-species, and to differentiate within more closely related groups such as the *aus* or *japonica* accessions. Each SNP probe was classified into three main groups; those that should discriminate between *indicas* and *japonicas,* within *japonicas,* or within *aus.* The SNP array was also designed so that there was an approximately even spread of markers of different categories across each genome (Fig. 1). Additional file 5: Table S3 (Online Resource 3) describes the classification of all probes used with their flanking sequences, SNP and physical position. Genomic DNA from each of the cultivars was extracted and 500 ng of each sample placed in 96 well plates used for the 384 SNP Illumina GoldenGate oligo pool assay (Illumina Inc.) using the BeadXpress platform, according to the manufacturer's protocol. Allele calls were performed using the “GenTrain” clustering algorithm available in Genome Studio v2011.1 (Illumina Inc.). Each SNP-call was checked manually in Genome Studio for quality and accuracy because rice is predominantly inbred, but the SNP detection algorithm used by Genome Studio was originally developed for out-breeders and by default searches for clusters of three SNP markers (two homozygotes and one heterozygote). Any allele calls below a threshold of 0.02 NormR were discarded.

### Data analysis

Poor quality SNP markers were excluded from the analysis by removing any markers where there was statistical evidence that plates varied in the amount of heterozygosity detected. This removed a total of 58 SNP markers as shown in Additional file 5: Table S3 (Online Resource 3). The MCMC (Markov Chain Monte Carlo) population analysis program “STRUCTURE” (Pritchard *et al.,*^2000^; Falush *et al.,*^2003^) was used to infer underlying population structure in the SNP data using an initial burn-in of 5,000 iterations, followed by a run length of 50,000 iterations. The population structure in the data was investigated using putative population ‘K’ values ranging from 2-10 (10 replicates per K value) with a STRUCTURE model including admixture and correlated allele frequencies. STRUCTURE Harvester (Earl and VonHoldt 2012) was used to establish an optimum K value from the results of the exploratory STRUCTURE analysis using the Evanno “Delta-K” method (Evanno *et al.,*^2005^). An optimum K value of 4 groups was established for Panel A (511 cultivars) as shown in Additional file 1: Figure S1 (Online Resource 4). The SNP data was then re-analysed using STRUCTURE with a putative K value of 4 groups, burn-in of 10,000 iterations and run length of 100,000 iterations to generate a STRUCTURE ‘Q’ matrix. Major modes in the STRUCTURE output were identified using CLUMPAK (Kopelman *et al.,*^2015^) and are presented in Additional file 2: Figure S2a (Online Resource 5).

Rice subpopulations (*indica, japonica* or *aus*) were assigned to each of the four groups identified by STRUCTURE, based on a number of ‘exemplar’ cultivars from the OryzaSNP set and Rice Diversity Panel 1 for which the rice group was known *a-priori* as presented in Additional file 3: Table S1 (Online Resource 1). Two of the groups identified by STRUCTURE contained exemplars from the *aus* rice group and were arbitrarily named *aus-1* and *aus-2*. Cultivars were only assigned to a group if the probability of their group membership determined by STRUCTURE was ≥ 80 %. Cultivars with < 80 % probability of a single group membership were classified as ‘*admix*’ unless their combined *aus* group memberships (*aus-1 + aus-2*) was ≥ 80 %. These cultivars were classified as *aus-admix*. The quantitative genetics program TASSEL (Bradbury *et al*., 2007) was used to perform Principal Component Analysis (PCA) of the SNP data, after filtering and imputation of missing SNPs using default parameters. The TreeBest program from “TreeFam” (Ruan *et al.,*^2008^) was used to obtain a 50 % consensus tree bootstrapped from 100 NJ (Neighbour-Joining) trees based on multi-FASTA alignments of SNPs. The NJ Trees were plotted using Dendroscope (Huson *et al.,*^2007^). The groups identified by STRUCTURE were used to assign coloured labels to groups on the PCA plots (Fig. 2) and NJ trees (Figs. 3 and 4). The Dendroscope nexml format files used to plot the NJ trees presented in Figs. 4 and 5 are provided as Additional file 6 and 7 in the supplementary material.

Prior population information about the exemplar cultivars was *not* used in the STRUCTURE model used to analyse Panel A (511 cultivars). However, cultivars with ≥ 80 % probability of single group membership identified in the analysis were used to create a training-set for analysis of Panel B (74 cultivars) with greater genetic diversity than the relatively small number of exemplar cultivars included in analysis of Panel A. Prior population information about the training set *was* included in the STRUCTURE model used to analyse Panel B.

## Additional files

Additional file 1: Figure S1. Optimum K value of 4 groups established for Panel A (511 cultivars). Results obtained from STRUCTURE were analysed by the Evanno ‘Delta-K’ method using STRUCTURE Harvester. (DOCX 59 kb) Additional file 2: Figure S2. Major modes detected by STRUCTURE. Modes for cultivars in (a) Panel A (511 AWD cultivars) and (b) Panel B (74 Indian cultivars) for K = 4 from the Evano method were obtained using CLUMPAK with search method LargeKGreedy, MCL cluster size threshold = 0.1 and cut-off = 0.50. (DOCX 62 kb) Additional file 3: Table S1. Accessions of panel A with STRUCTURE group membership and PCA results. (XLSX 97 kb) Additional file 4: Table S2. Accessions of Panel B with STRUCTURE group membership. (XLSX 18 kb) Additional file 5: Table S3. SNPs in the 384 Illumina GoldenGate array (from Rice Diversity website http://ricediversity.org/). (XLSX 54 kb) Additional file 6: Dendroscope nexml format file used to plot the Neighbour-Joining tree shown in  Fig 4. (NEXML 453 kb) Additional file 7: Dendroscope nexml format file used to plot the Neighbour-Joining tree shown in  Fig 5. (NEXML 400 kb)
