# Supplementary figures and images for: Assessing the genetic diversity of rice originating from Bangladesh, Assam and West Bengal
Source: Rice (N Y). 2015 Dec 1;8:35. doi: 10.1186/s12284-015-0068-z (PMC4667538; doi:10.1186/s12284-015-0068-z)

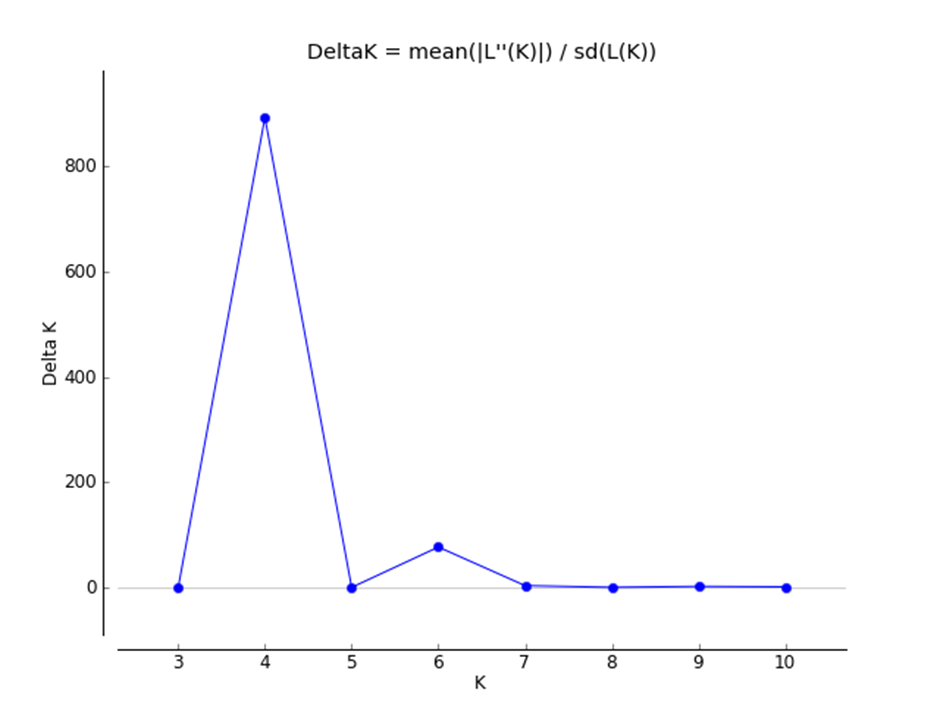

Supplement: Additional file 1: Figure S1. — Optimum K value of 4 groups established for Panel A (511 cultivars). Results obtained from STRUCTURE were analysed by the Evanno ‘Delta-K’ method using STRUCTURE Harvester. (DOCX 59 kb) [file 12284_2015_68_MOESM1_ESM.docx]

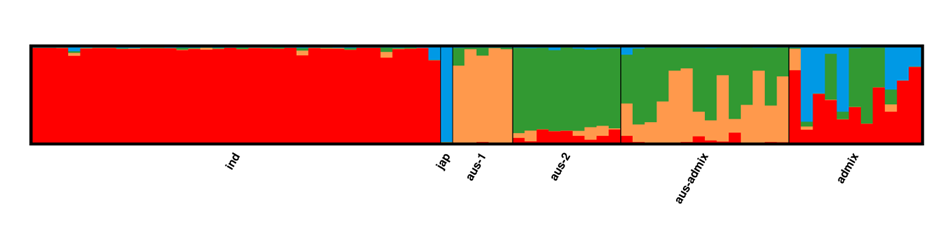


(a)


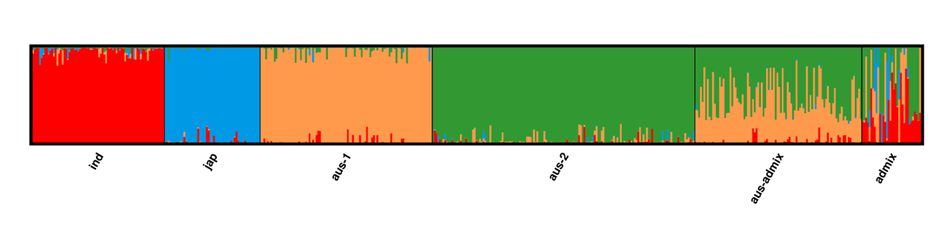


(b)

Supplement: Additional file 2: Figure S2. — Major modes detected by STRUCTURE. Modes for cultivars in (a) Panel A (511 AWD cultivars) and (b) Panel B (74 Indian cultivars) for K = 4 from the Evano method were obtained using CLUMPAK with search method LargeKGreedy, MCL cluster size threshold = 0.1 and cut-off = 0.50. (DOCX 62 kb) [file 12284_2015_68_MOESM2_ESM.docx]
